# Supplementary material for: Selective Adsorption of Aqueous Diclofenac Sodium, Naproxen Sodium, and Ibuprofen Using a Stable Fe3O4–FeBTC Metal–Organic Framework
Source: Materials (Basel). 2021 Apr 28;14(9):2293. doi: 10.3390/ma14092293 (PMC8124272; doi:10.3390/ma14092293)
Supplement: Supplementary file 1 [file materials-14-02293-s001.zip › materials-1170842-supplementary.pdf]

materials

Article

Selective Adsorption of Aqueous Diclofenac Sodium, Naproxen Sodium, and Ibuprofen Using a Stable Fe_3O_4–FeBTC Metal–Organic Frameworka

Aldo Arturo Castañeda Ramírez [1,*], Elizabeth Rojas García [2], Ricardo López Medina [3], José L. Contreras Larios [3], Raúl Suárez Parra [4] and Ana Marisela Maubert Franco [1]

1 Materials Chemistry , Basic Sciences, Metropolitan Autonomous University -Azcapotzalco, 02200 Mexico City, Mexico; amf@azc.uam.mx
2 Process Engineering and Hydraulics, Basic Sciences, Metropolitan Autonomous University -Iztapalapa, 09340 Mexico City, Mexico; erg@xanum.uam.mx
3 Energy, Basic Sciences, Metropolitan Autonomous University -Azcapotzalco, 02200 Mexico City, Mexico; rilome@correo.azc.uam.mx (R.L.M.), jlcl@azc.uam.mx (J.L.C.L.)
4 Institute of Renewable Energies, National Autonomous University of Mexico , 62580 Morelos, México; rsp@ier.unam.mx
* Correspondence: al2151801162@azc.uam.mx, Tel.: +52-5571203078

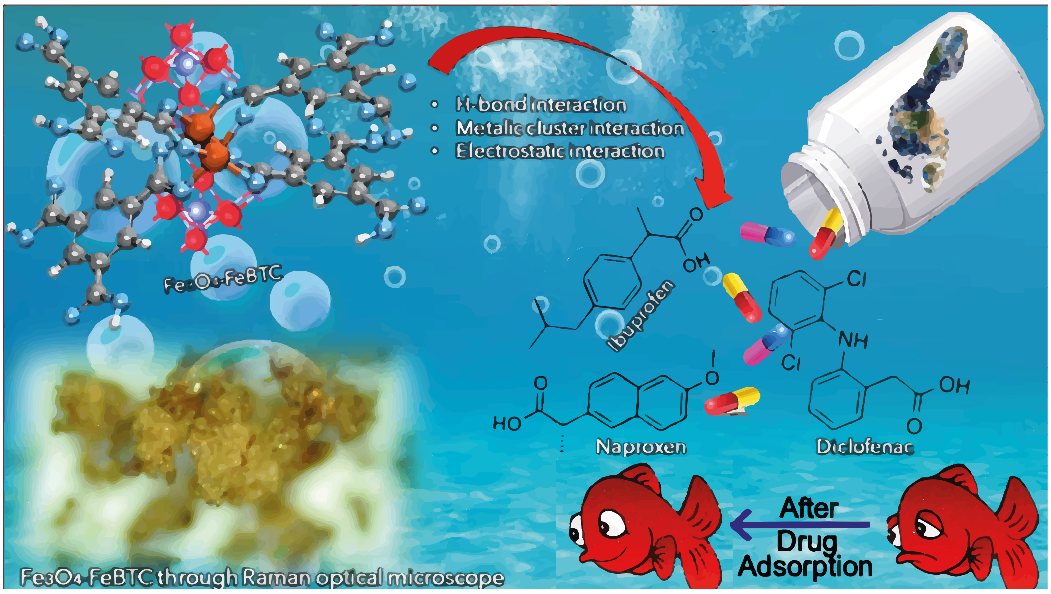

Figure 1. Fe_3O_4-FeBTC MOF being used to remove diclofenac sodium, naproxen sodium, and ibuprofen from water-inset shows a view through a Raman microscope.

The magnetite diffraction pattern (Figure S2) shows seven main reflections, 2θ = 19, 30, 36, 44, 54, 57, and 63°, corresponding to planes (220), (311), (400), (422), (511), and (440), respectively, associated with a simple cubic system of Fd-3m spatial group (JCPDS PDF19-0629). The average crystallite size of magnetite was 19.3 nm, which was calculated using the Scherrer equation.

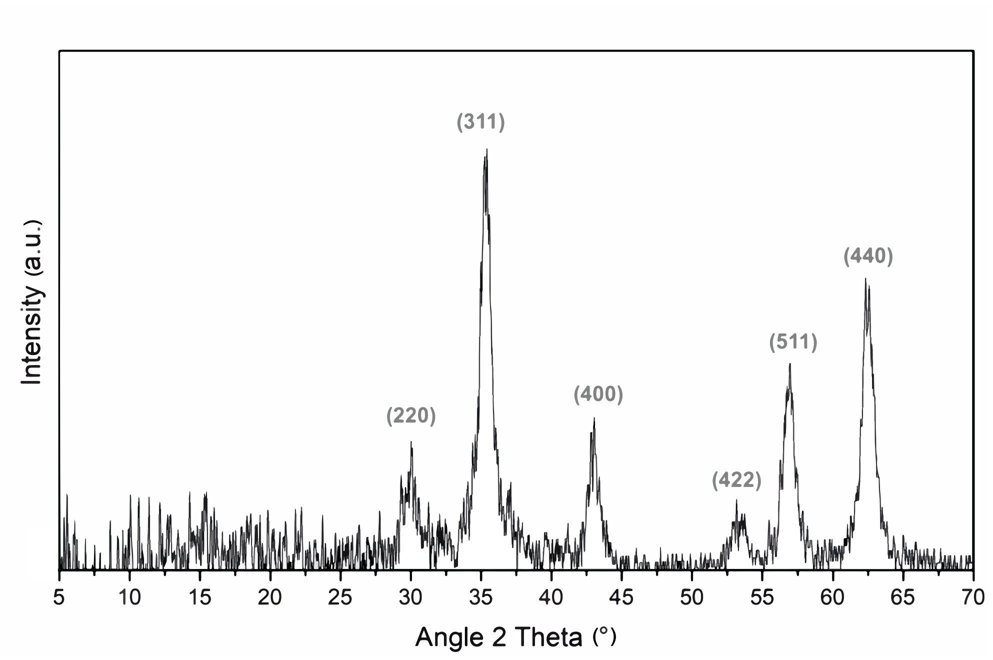

Figure 2. XRD patterns of magnetite nanoparticles.

It is possible to see a sharp peak at about 570 cm−1 (Figure S3), characteristic of Fe–O bond absorption in the most representative magnetite band. The maximum band presence at 3500 cm^{-1} is typical of H–O bonds related to the material's humidity. The C–O bond's stretching vibration at 1600^{-1} is characteristic of small impurities from ethylene glycol.

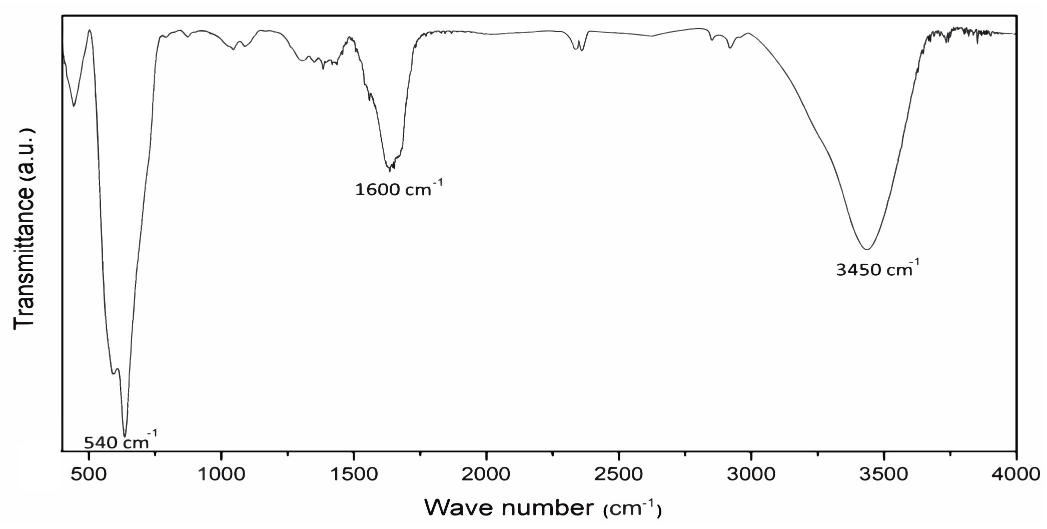

Figure 3. Fourier transform infrared spectroscopy spectra of magnetite.

The results of Raman spectroscopy (Figure S4) show the spectrum characteristics of magnetite nanoparticles, where the top band located at 661 cm-1 refers to the modes of vibration stretching of oxygen bound with iron (II) and (III) along the entire crystal lattice. The second most important band is located at 310 cm^{-1}, and the ones situated below 500 cm^{-1} are represented by the phononic activity caused by the dispersion of light after polarization of the material.

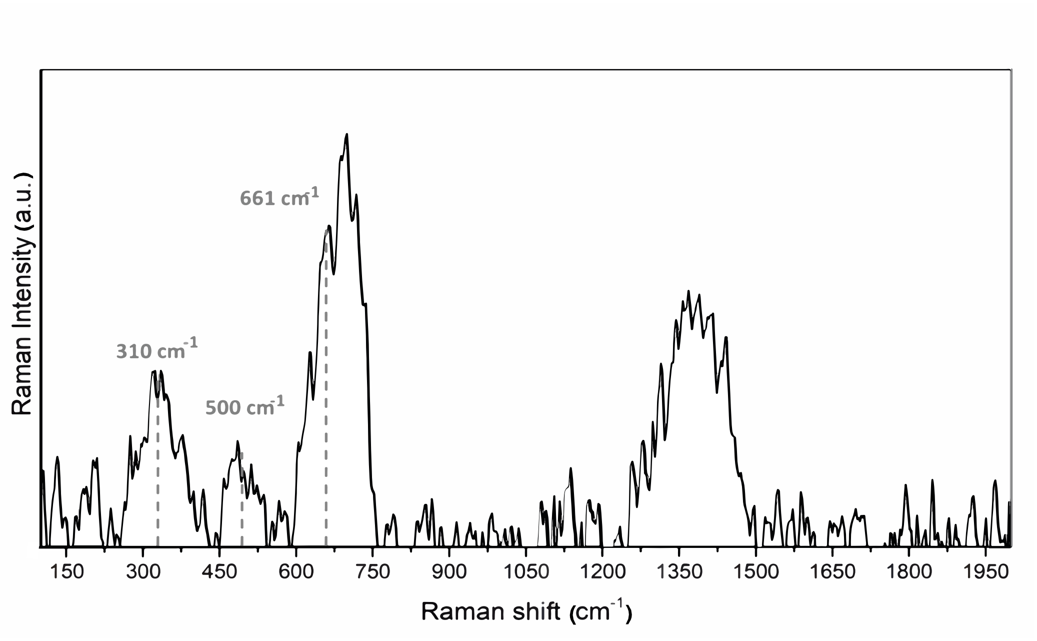

Figure 4. Raman spectra of magnetite.

Figure S5 shows the materials through a Raman microscope. The images do not show significant differences between FeBTC and Fe_3O_4-FeBTC regarding superficial morphology.

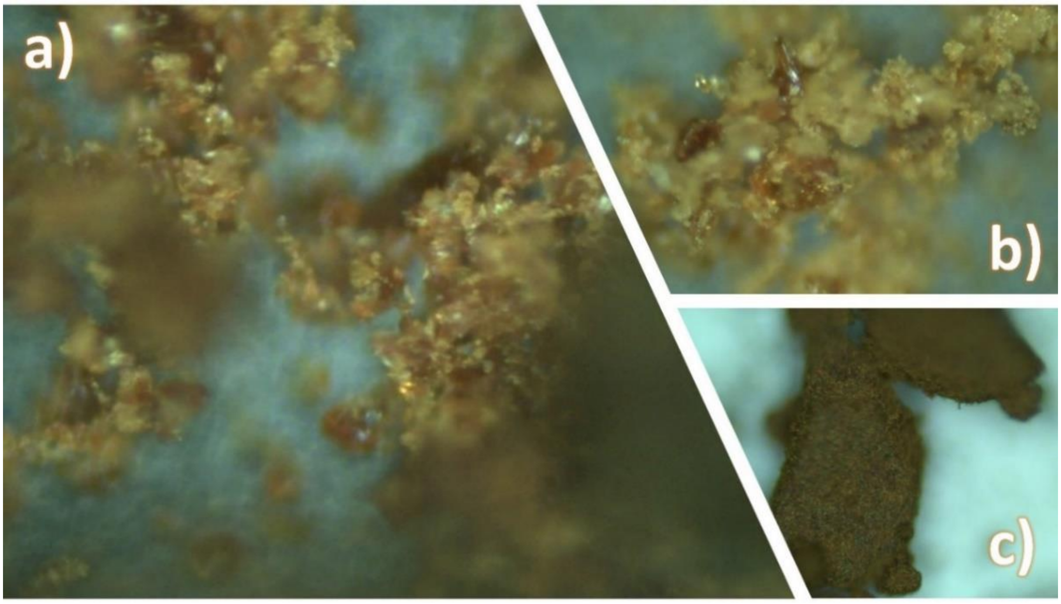

Figure 5. Raman micrographs of (a) Fe_3O_4-FeBTC, (b) FeBTC, and (c) magnetite.

Scanning electron microscopy (Figure S6) was performed to obtain information on the particles' morphology and the apparent size of their average particle. The FeBTC observed in Figure 6a–c presents particles that form bars and cubes, with a non-homogeneous and very well-defined population typical of this material, referred to in previous works.

Characterization by EDS was carried out to corroborate the presence of representative elements of the material. Although this study was merely semi-quantitative, it approximates the proportion of elements present in the material, comparing stoichiometry. In FeBTC, only the elements analysis that is part of the MOF (Fe, O, and C) with some

impurities below 1% are presented, which adjust quite well to the material's stoichiometry after explored three regions of the material.

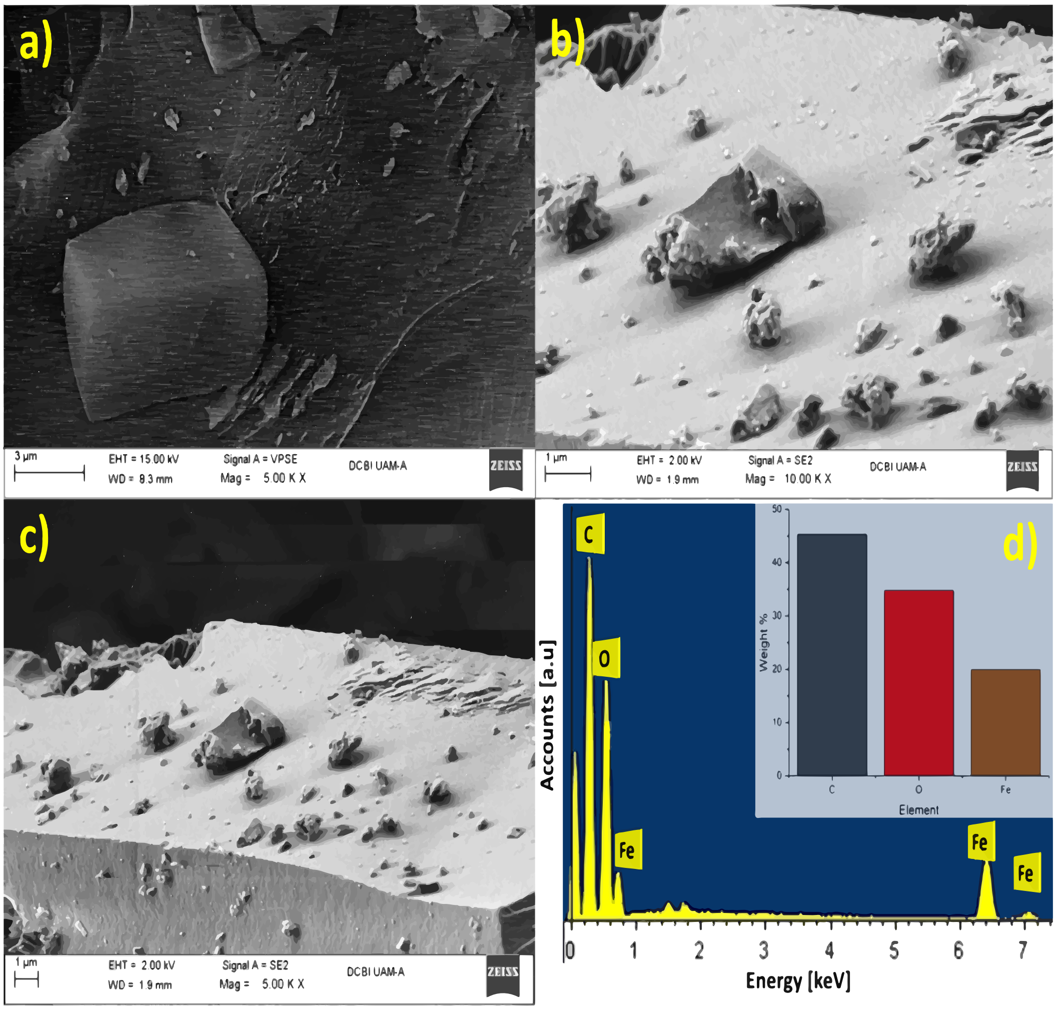

Figure 6. Scanning electron microscopy of FeBTC: (a) scanning at 5 Kx by variable pressure, (b) Scheme 5. kx with secondary electrons, (c) scanning at 10 kx with secondary electrons, and (d) by dispersive energy (results).

Scanning electron microscopy of the Fe_3O_4-FeBTC composite MOF (Figure S7) shows the presence of particles that tend to form bars, like the FeBTC MOF in its pristine state, and they do not have a well-defined morphology or a specific size (Figure S6a–c). On the other hand, EDS characterization shows small increases in the populations of oxygens and irons, which is justified by magnetite presence (Figure S6d).

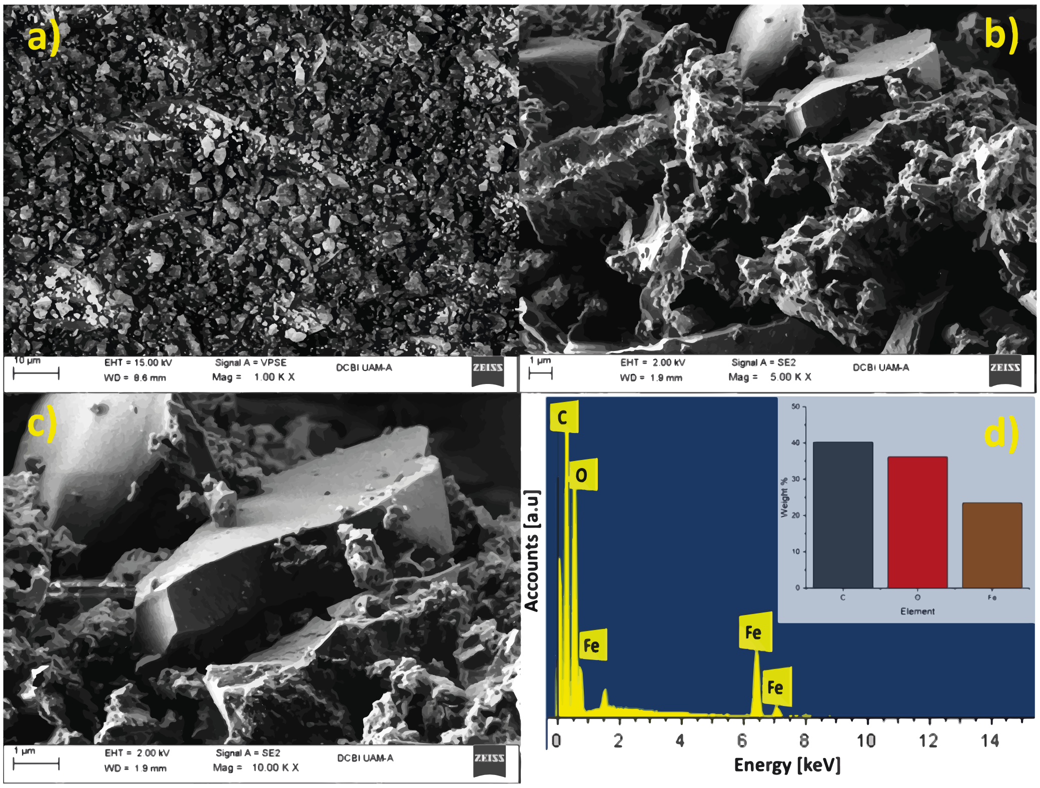

Figure 7. Scanning electron microscopy of Fe_3O_4-BTC: (a) scanning at 5 Kx by variable pressure, (b) scanning at 5 kx with secondary electrons, (c) scanning at 10 kx with secondary electrons, and (d) by dispersive energy (results).

The materials' thermal stability was evaluated using in situ Raman spectroscopy using a Linkam cell reactor (Figure S8). The procedure consisted of increasing the temperature until the decomposition temperature of MOF with nitrogen flow as a treatment gas. The results reveal that the band to 500 cm^{-1} correspondings to Fe–O vibrations tended to disappear with increasing temperature.

The incorporation of magnetite provided FeBTC with greater stability (523 K, 30 K more than FeBTC). Because—as the characterization by this same technique shows—the magnetite was incorporated in the most vulnerable sites of the MOF in such a way that it could reinforce the structure (Figure S8).

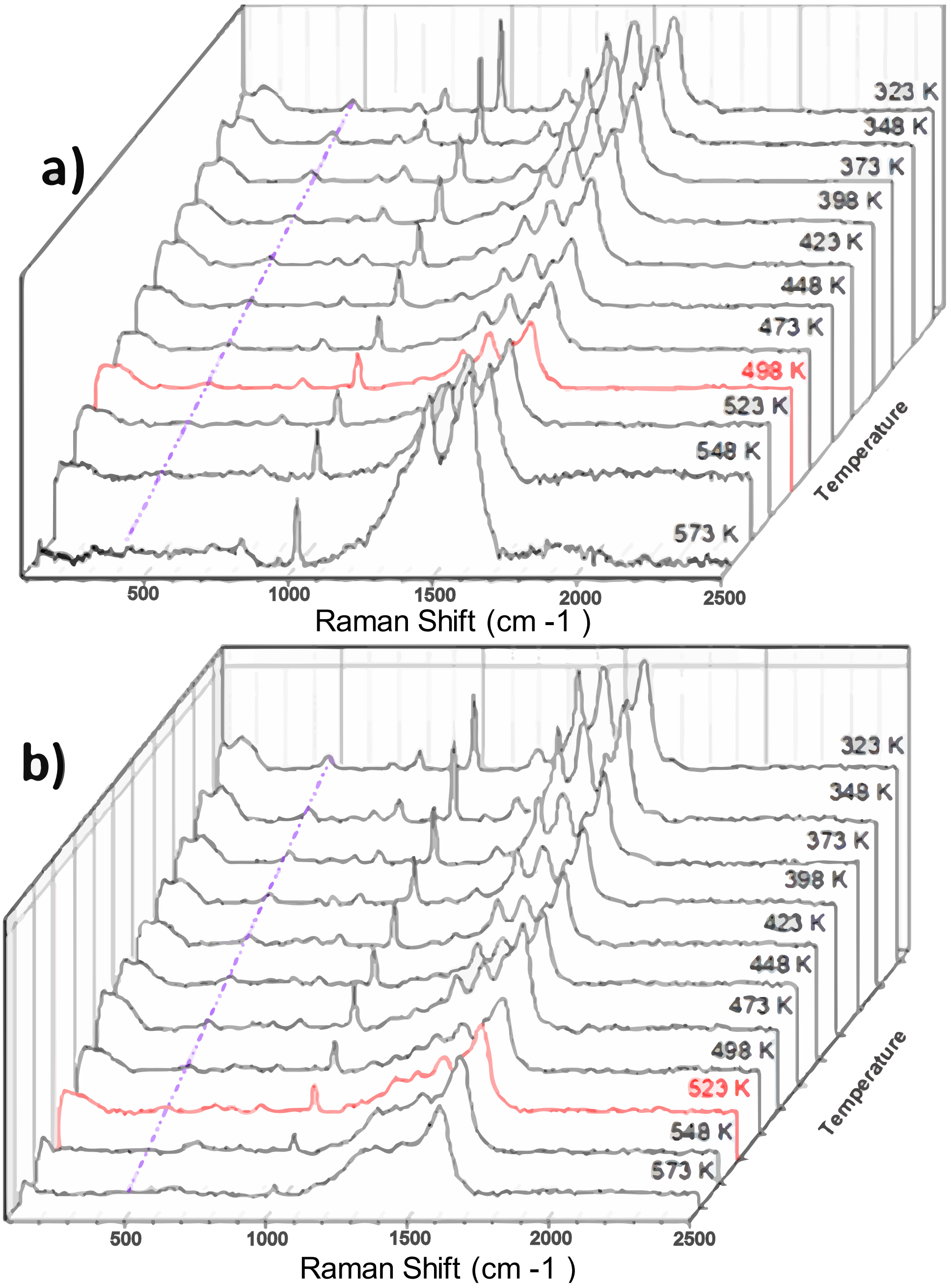

Figure 8. Thermal stability followed by on-site Raman spectroscopy of (a) FeBTC and (b) Fe_3O_4-FeBTC.

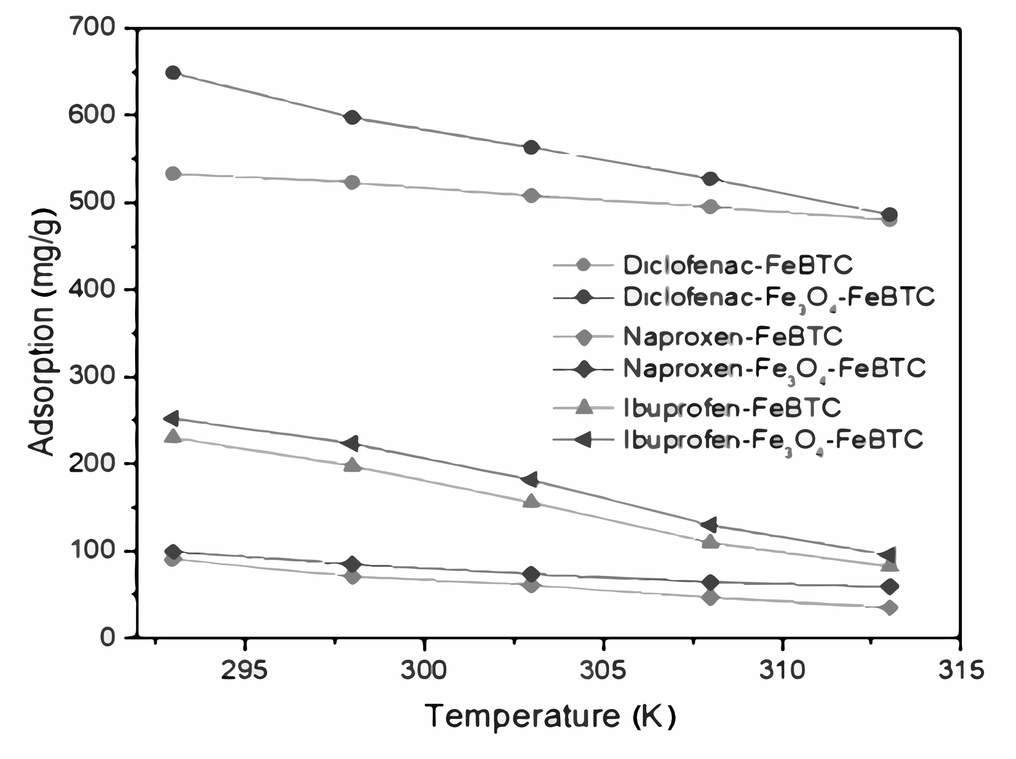

Figure 9. Influence of temperature on the adsorption of drugs on FeBTC and Fe_3O_4-FeBTC.

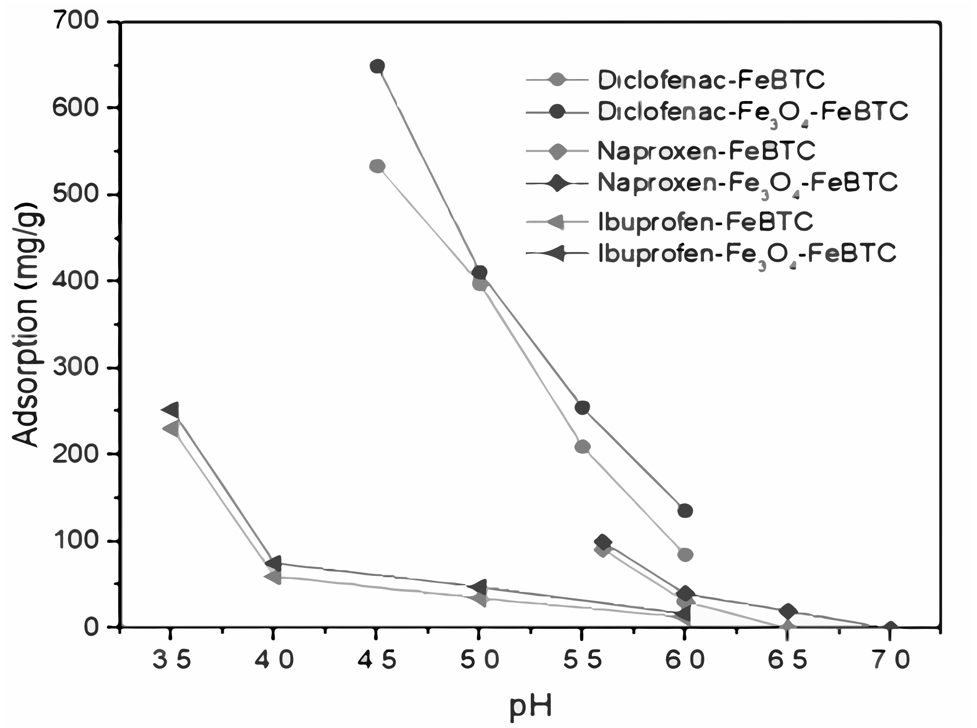

Figure 10. The PH effect on the drugs adsorption on the MOF´s at the constant conditions of 3 mg adsorbent concentration and 293.15 K. .
